# Innovating Stroke Recovery: A Systematic Review of Virtual Reality in Cognitive Rehabilitation

**DOI:** 10.7759/cureus.95573

**Published:** 2025-10-28

**Authors:** Arasi Pearia Anadachee, Riya Patil, Kshitija Gajadhur, Hutesh Singh, Milakshna Devi Chuckowry, Ashima Elsa Philip, Indrajit Banerjee

**Affiliations:** 1 Internal Medicine, Sir Seewoosagur Ramgoolam Medical College, Belle Rive, MUS; 2 Pharmacology, Sir Seewoosagur Ramgoolam Medical College, Belle Rive, MUS

**Keywords:** montreal cognitive assessment (moca), physical medicine and rehabilitation, post-stroke cognitive impairment, post stroke recovery, virtual reality (vr)

## Abstract

Virtual reality (VR), a surging therapeutic tool especially in stroke rehabilitation, has emerged as a powerhouse for VR-based rehabilitation in stroke patients, which is not only restricted to motor dysfunction but also encompasses cognitive impairment. Given the central role of cognition in activities of daily living, identifying effective interventions to address post-stroke cognitive impairment is critical. Hence, this systematic review aims to evaluate the effects of VR on cognitive function in stroke patients.

An extensive search was conducted on PubMed, Cochrane Central Register of Controlled Trials (CENTRAL), Turning Research into Practice (TRIP), Google Scholar, Science Direct, Web of Science, and Scopus databases, with Medical Subject Headings (MeSH) terms “virtual reality”, “cognitive function”, and “stroke” for relevant articles. Eligible randomized controlled trials (RCTs) published from January 2019 to August 2025 were reviewed. The Cochrane risk-of-bias tool (RoB 2) was used for methodological appraisal. The review followed Preferred Reporting Items for Systematic Reviews and Meta-Analyses (PRISMA) guidelines to ensure transparency and compliance with standard guidelines.

Out of 91,713 articles initially identified, 9 RCTs met the inclusion criteria and encompassed a total of 601 stroke patients, comprising 53% females. The primary outcome was global cognitive function assessed by the Montreal Cognitive Assessment, which was statistically significant in 78% of the articles following VR-based interventions compared to conventional therapy. The secondary outcomes measured included: mental health by Mini-Mental State Examination (n=2), Beck Depression Inventory-II (n=1), Hospital Anxiety and Depression Scale (n=1) or Hamilton Anxiety Rating Scale (n=1); disability and autonomy measured by Modified Barthel index (n=1) and Functional Independence Measure (n=2); and quality of life (QoL) assessed by Short form-12 Health Survey (n=1) and EuroQoL (n=1). Statistical improvement was observed in only two studies for mental health and in one for QoL.

VR-based cognitive rehabilitation is associated with meaningful improvements in global cognitive function. The findings of this review support integration of VR as a complementary tool in the multidisciplinary rehabilitation of stroke survivors with cognitive impairments.

## Introduction and background

Over the past few decades, stroke has emerged as a major contributor to long-term disability worldwide, clearly demonstrated by the Global Burden of Disease estimates, as stroke currently accounts for approximately 4-5% of global disability adjusted life years (DALYs)[1-2]. Disability is tackled by rehabilitation pathways that mostly cater to motor recovery, hence insufficiently addressing functions such as cognition, depression, and fatigue [3]. An oversight that proves devastating when non-motor impairments especially cognition can prove as socioeconomically debilitating as their motor counterparts, notably demonstrated by 40% of stroke patients who develop post-stroke cognitive impairment within 4 years of stroke, a condition associated with an increased number of visits to the general practitioner, and/or neurologist prolonged hospital stays and in many cases loss of employment [4-5].

In recent years, virtual reality(VR) based rehabilitation has emerged as a promising alternative with its engaging task-oriented environment that stimulates neuroplasticity while enhancing patient motivation [6-7]. Nevertheless, it is to be remarked that despite this dual improvement potential, most of the previous VR platforms were primarily designed to focus on motor function improvement and, it is only now that with increasing number of randomized controlled trials (RCTs) investigating cognitive gains via VR therapy, that a systematic review on the subject has become more viable [6, 8-10]. Previous systematic reviews and meta-analyses on the subject have, to date, largely relied on pilot studies or a limited pool of RCTs, which have precluded a comprehensive evaluation of cognitive outcomes or the influence of baseline characteristics, such as time since stroke or side of brain lesion [9-10]. The recent proliferation of high-quality RCTs featuring extended follow-up periods and detailed baseline data, including time post-stroke, lesion laterality, and lesion type, allows us to overcome these limitations and enables a more robust analysis of the topic.

Accordingly, this systematic review has as its main aim to evaluate the impact of VR-based interventions on global cognitive function in adult stroke survivors, as measured by the Montreal Cognitive Assessment (MoCA), which has been proven as an adequate tool used for assessment of general cognitive function, specifically in stroke patients [11]. This systematic review also compared the relative effectiveness of immersive versus non-immersive VR applications and examined secondary outcomes related to functional independence, mental health, and quality of life (QoL). All done, along with a methodological assessment of the RCTs that ensured a solid, evidence-based evaluation of our primary aim: the evaluation of the effectiveness of VR-based cognitive rehabilitation in stroke patients assessed by MoCA.

## Review

Methodology

**Figure 1:**
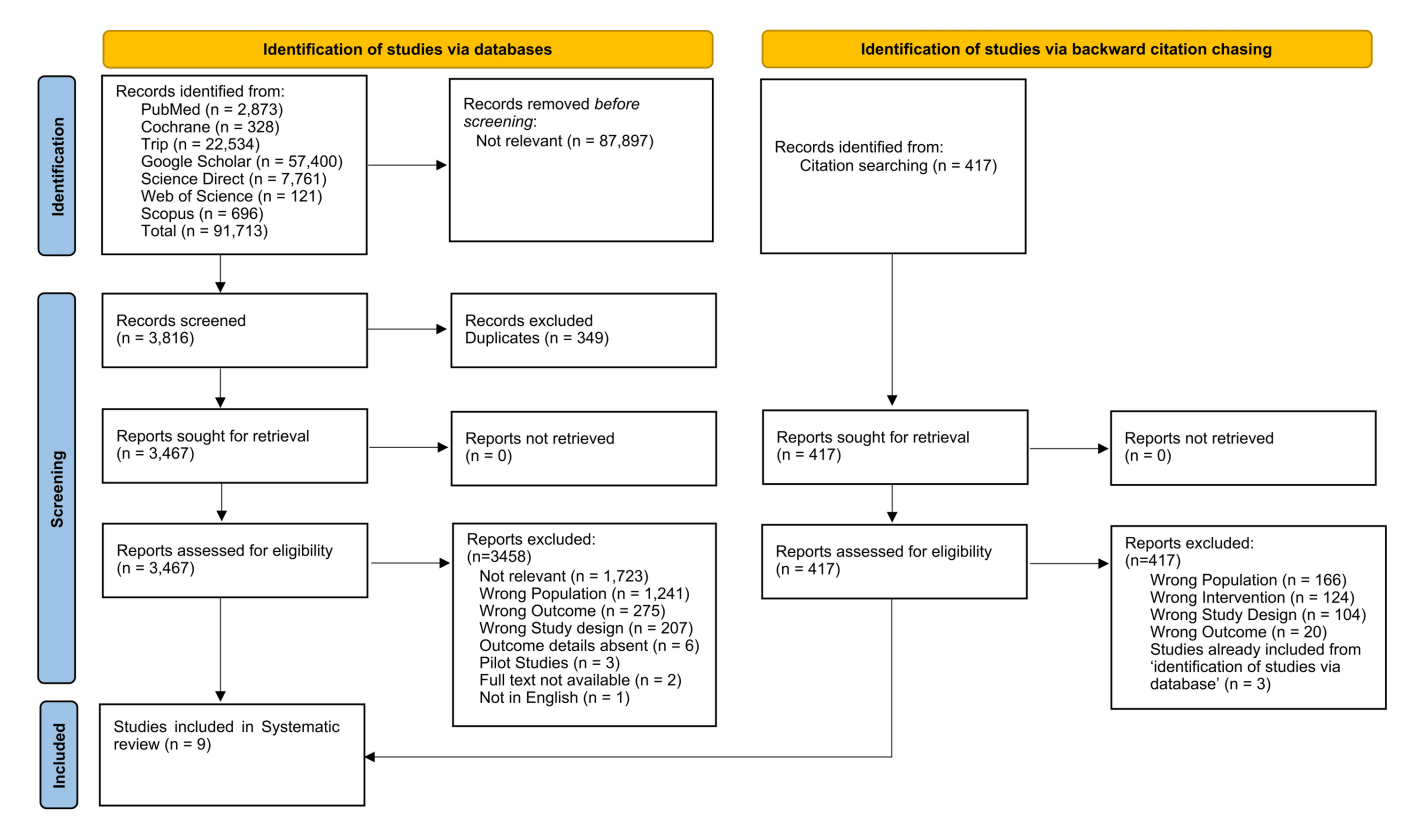
PRISMA 2020 Flow Diagram

A systematic review was conducted under the Preferred Reporting Items for Systematic Reviews and Meta-Analyses (PRISMA) 2020 guidelines (Figure 1) [12].

Search Strategy

A comprehensive literature search was performed on the following medical databases: PubMed, Cochrane Central Register of Controlled Trials (CENTRAL), Turning Research into Practice (TRIP), Google Scholar, Science Direct, Web of Science, and Scopus. The Medical Subject Headings (MeSH) terms and Boolean operators used were Stroke AND Cognitive training AND Virtual reality.

Inclusion Criteria

The current review included completed RCTs published between January 2019 and August 2025, written in English. Eligible studies must involve stroke patients and examine the effect of VR interventions aimed at improving cognitive function. Cognitive domains of interest were - attention, memory, language, problem-solving, executive function, reasoning, and decision-making. Additionally, included studies must utilize cognitive outcomes using the MoCA scale.

Exclusion Criteria

Non-randomized controlled trials (non-RCTs), cohort studies, case-control studies, cross-sectional studies, abstracts, case studies, editorials, viewpoints, case series, commentaries, expert reviews, and letters to the editor were excluded from this study. Studies published outside the time frame of January 2019 to August 2025, or in languages other than English, were excluded. Additionally, studies involving populations other than stroke patients, those that have not used VR as a part of intervention, lack an assessment of cognitive function, or do not utilize the MoCA scale were also not considered eligible for inclusion.

Study Identification and Selection

Blinded screening of RCTs was done independently by four authors (MC, AA, RP, KG) using the Rayyan platform, based on titles and abstracts [13]. This was followed by reviewing the full texts of the eligible articles. Only those articles that met the predefined selection criteria were included, and backward citation chasing was done by AA, MC, and HS to get the final list of selected RCTs.

Data Extraction

The selected RCTs were analyzed, and a data synthesis table was made using Google Sheets. The extracted data included study characteristics (title, authors, year, country, duration), participant characteristics (age, sample size, size of intervention and control groups), type of stroke, intervention details including type of VR used, outcomes measured, pre- and post-intervention MoCA scores, and potential limitations of the study.

Risk of Bias Assessment

Risk of bias of the included studies was assessed qualitatively by three authors (KG, AA, and RP) using the Cochrane risk of bias tool for randomized trials (RoB 2) [14]. The following criteria were considered: randomization procedure, concealment of allocation, blinding of the outcome assessor, completeness of outcome data, and risk of selective reporting. The selected RCTs were classified accordingly as low risk, high risk, or of some concern. The data were then transferred to the Robvis visualization tool for generating traffic light plots and weighted bar plots [15].

Results

The initial database search yielded 91,713 results, among which 87,897 results were removed as they fell outside the scope of the research objective. After removal of 349 duplicates, the remaining 3,467 records were retrieved for screening and assessed based on title and abstract. Of these, 3,458 articles were excluded for the following reasons: irrelevance, incorrect study population, outcome not aligned with the objective of the systematic review, inappropriate study design, missing data, being pilot studies, no full text available, and absence of an English version of the article (Figure 1). Subsequently, 9 RCTs were deemed eligible to be included. A total of 417 records were identified and retrieved through backward citation chasing. All 417 articles were rejected due to the factors outlined below: wrong population, wrong intervention, wrong study design, wrong outcome, and studies already included during the initial database search. Ultimately, 9 RCTs satisfied the inclusion criteria and were considered suitable for the systematic review. Figure 1 highlights the whole screening process with an initial 91,713 count, which narrowed down to nine during final selection.

As shown in Table 1, the nine RCTs were conducted across countries such as Europe and Asia between 2019 and 2025, involving both acute and chronic stroke populations with sample sizes ranging from 31 to 126 participants and ages spanning from 43 to 77 years. Time from stroke onset to intervention varied from 9 days to 45 months. Intervention groups received VR-based therapy, which was non-immersive (n=6), immersive (n=1), semi-immersive (n=1), or mixed (n=1), and was usually combined with standard rehabilitation or robotic therapy in one of the studies, while control groups typically received conventional rehabilitation training(CRT) for cognitive functions.

**Table 1:** Study Characteristics and Patients’ Demographics *names of the programs used in the study.

Author, Year	Country of Study	Study Design	Duration of Study	Targeted Population	Eligibility Criteria for Patient Selection	Strategy Carried Out in Intervention Group (IG)	Strategy Carried Out in Control Group (CG)	Type of Virtual Reality (VR)	Sample Size			Age, Years ± Standard Deviation (SD)		Duration Between Last Onset of Stroke And Start of Intervention	
									IG	CC	Total	IG	CG	IG	CG
Baltaduonienė et al., 2019 [16]	Lithuania	RCT	36 months	First-time patients with ischemic stroke (10-14 days following a stroke)	First-time ischemic stroke patients Participants having a Barthel index (BI) of 50–65 points Participants with a mini-mental state examination (MMSE) score of ≥11 points Consenting participants with no visual or hearing impairment	Conventional Rehabilitation Training (CRT) and VR rehabilitation training using SeeMe®	CRT with “pencil–and–paper” cognition training tasks	Non-Immersive	42	42	126	74.33 ± 10.27	69.71 ± 11.67	10-14 days	10-14 days
Oh et al., 2019 [17]	Republic of Korea	RCT	6 weeks	Chronic Stroke Patients	Patients with their first ever episode of stroke as indicated by brain CT (computed tomography) or MRI (Magnetic resonance imaging) Patients who were evaluated for at least 6 months following onset of stroke Age of participants: 20-85 years Patients with one-sided paralysis or paresis, with Fugl-Meyer Assessment for Upper Extremity (FMA-UE) score >18 Motivation and cooperation of patient participating in the study	Combination of VR and real instrument training	CRT	Non-Immersive	17	14	31	57.4 ± 12.2	52.6 ± 10.7	During the initial 6 months of the onset of the first episode of stroke	During the initial 6 months of onset of first episode of stroke
Manuli et al., 2020 [18]	Italy	RCT	9 months	Chronic stroke patients (Ischemic and hemorrhagic)	Chronic stroke patient Ability to sit without support for at least 1 minute and up to 30 minutes in total Starting MoCA score: 16-24	Robotic rehabilitation and VR using Lokomat-Pro	CRT	Non-immersive	30	30	90	48.0 ± 12.1	43.1 ± 9.7	4.5 ± 1.5 months	4 ± 2 months
Mao et al., 2020 [19]	China	RCT	2 months	Stroke patients (infarcts and hemorrhages)	Patients with their first occurrence of stroke as demonstrated by brain MRI, stable vital signs, unilateral paralysis and impairment of cognitive function Illness course within 3–9 months Age group: 40-80 years Written informed consent and willingness to participate in study Based on the Edinburgh Handedness Inventory, selected participants must be right-handed Patient having more than 9 years of formal education Participant with an upper extremity score of Brunnstrom stage II-IV MoCA: more or equal to 15	Rehabilitation training of the upper limb with Schulte Grid experiment and mirror neuron-based cognitive training by MNST V1.0*	Rehabilitation training of the upper limb with the Schulte Grid experiment	Semi-immersive VR or Video-based action observation therapy (AOT)	30	30	60	54 ± 7	57 ± 6	3-9 months	3-9 months
Faria et al., 2020 [20]	Portugal	RCT	24 months	Chronic Stroke Patients (Ischemic and hemorrhagic)	Patients aged 75 years or less Participants had their first episode of stroke and were in chronic phase of stroke Absence of hemi-spatial neglect assessed clinically Participant able to sit down Participant having at least 2 years of formal education Motivated to take part in the study	Task generator paper and pencil tasks set in a virtual world in Reh@City v2.0*	Task generator- personalized paper and pencil task in PDF format, which was printed and used	Non-immersive	14	18	32	59.14 ± 11.81	65.00 ± 6.20	45.93 ± 43.56 months	21.33 ± 12.88 months
Xuefang et al., 2021 [21]	China	RCT	24 months	Chronic Stroke Patients (Ischemic and hemorrhagic)	Patients with their first-ever episode of stroke, which met the established diagnostic guidelines for stroke The selected individuals had ischemic stroke, which was confirmed by radiological imaging such as MRI or CT Stable without any progression of disease for more than 2 days No current/ past mental illness or dysfunction Informed consent of patients and responsible parties	Motion observation therapy using VR equipment and CRT	CRT	Non-immersive	59	59	118	62.7 ± 1.6	65.3 ± 1.4	17.8 ± 0.3 days	19.5 ± 0.4 days
Gueye et al., 2021 [22]	Czech Republic	RCT	54 months	Acute Stroke Patients (Ischemic and hemorrhagic)	Patients with their first episode of acute stroke whereby the interval between stroke and start of intervention did not exceed 30 days Ability to cooperate Presence of deficit in the upper limb following stroke	VR (Armeo Spring Device*) and daily rehabilitation sessions	Conventional physiotherapy and daily rehabilitation sessions	Non-Immersive	25	25	50	66.56 ± 12.26	68.12 ± 11.97	14.88 ± 6.45 days	16.4 ± 7.25 days
Chatterjee et al., 2022 [23]	United Kingdom	RCT	13 months	Unilateral Stroke	Patient being more than 18 years of age Unilateral confirmed stroke within past 1 day to 3 weeks, which resulted in cognitive function impairment	VR-based cognitive treatment using VIRTUE* with usual care	Sham VR treatment with usual care	Immersive	30	10	40	77.5 ± 13.5	63 ± 26.5	9.5 ± 4.75 days	9 ± 8.25 days
Maggio et al., 2025 [24]	Italy	RCT	14 months	Chronic Stroke Patients (Ischemic and hemorrhagic)	Participant diagnosed with either chronic hemorrhagic or chronic ischemic stroke Age: 18-80 years MoCA score greater than 18 Participants possessed the cognitive and physical capacities to comprehend and adhere to instructions, and to engage in the rehabilitation processes	Cognitive training using VR Rehabilitation System (VRRS)	CRT with paper-based worksheets containing puzzles and memory tasks	Both non-immersive and immersive (depending on the type of task and intended cognitive outcome)	27	27	54	56.0 ± 8.55	53.8 ± 11.5	At least 6 months	At least 6 months

As observed from Table 2, the MoCA was the tool used to evaluate cognitive function in the selected articles. The results of the studies were reported as showing statistically significant improvement in cognitive functions between IG and CG (n=7), having no significant difference (n=1), and having observed an improvement in cognition in both IG and CG; however, there was no mention of the difference between the two groups (n=1). The magnitude of cognitive improvement was generally greater in studies employing immersive or semi-immersive VR systems. Common limitations included small sample sizes, short intervention durations, lack of long-term follow-ups, and limited generalization due to single-center designs.

**Table 2:** Outcome Measurement, Results, and Conclusion; Limitations of Study FIM: Functional Independence Measure

Author, Year	Measured Outcomes	Tools used to Measure Cognitive Function	Number of Outcomes Analyzed		MoCA Score before Study ± SD		MoCA Score after Study ± SD		Conclusion of Study	Limitations of Study
			IG	CG	IG	CG	IG	CG		
Baltaduonienė et al., 2019 [16]	Cognitive function	MMSE MoCA-LT (MoCA in Lithuanian language)	40	40	19.93 ± 4.04	18.08 ± 4.49	23.25 ± 3.92	21.03 ± 4.85	A greater improvement was seen in the cognitive function of the patients using VR during conventional rehabilitation therapy compared to those using conventional rehabilitation therapy alone.	Only general cognitive function was evaluated. The authors suggested that a better analysis could be made if various domains of cognitive function were to be assessed. Subjects exhibited low levels of confidence at the beginning of the trial, as they believed they would encounter difficulties while performing the required tasks. The Study did not evaluate how adaptable the intervention would be for people representing different demographic groups.
Oh et al., 2019 [17]	Cognitive function Muscle tone/spasticity Hand and finger coordination Upper limb coordination and dexterity Motor function and muscle strength	K-MMSE (Korean language MMSE) K-MoCA (MoCA in Korean language)	17	14	22.7 ± 4.2	24.4 ± 4.1	24.5 ± 4.2	26.1 ± 3.4	Cognitive performance improved in the intervention and control groups. Nonetheless, there was no mention of the significant difference between the two groups.	Small sample size, Heterogeneous sample was used. Long-term outcomes were not assessed.
Manuli et al., 2020 [18]	Cognitive function Frontal abilities Attention process, attention shifting, and visual research ability Mood evaluation Quality of Life Disability level of the participants Degree of independence while performing activities of daily living	FAB (Frontal Assessment Battery) MoCA, FIM-COGN (functional independence measure-cognition), TMT-A (Trail making test part A), TMT-B (Trail making test part B), VS (visual search), WEIGL (Weigl test)	30	30	21.8 ± 2.7	23.4 ± 2.4	26 ± 2.5	24.3 ± 2.3	VR combined with robotic treatment showed better changes in cognitive ability recovery in chronic stroke participants.	Assessment was conducted over a short-term period; hence, long-term benefits and disadvantages were not properly assessed. Single-center study - Generalization of study not possible. Accessibility and general use of VR and robotic devices could be limited due to their high costs.
Mao et al., 2020 [19]	Cognitive function Upper extremity motor function	MoCA Reaction time WCST (Wisconsin Card Sorting Test)	30	30	22 ± 6	22 ± 5	28 ± 2	24 ± 4	Combined use of MNS-based (Mirror Neuron System- based) and conventional rehabilitation has proven to contribute to promoting the level of cognitive performance.	Assessors were not blinded during the study.
Faria et al., 2020 [20]	Overall level of cognitive ability, Attention, Memory, Executive functions, Language	MoCA TMT-A TMT-B WMS-III (Verbal Paired Associates from the Wechsler Memory Scale III) WAIS-III (Wechsler Adult Intelligence Scale III)	14	18	23	21	25	21	A positive response was seen in general cognitive function with the use of Reh@City v2.0 in the rehabilitation protocol of stroke patients.	Many patients were lost at follow-up. The IG group used their paretic arm for the study, while in the CG, subjects used their healthy arm during the study.
Xuefang et al., 2021 [21]	Cognitive Function: Ability of Daily Living Impairment caused by Stroke	MoCA	59	59	18.4 ± 3.2	18.3 ± 2.3	26.8 ± 3.4	21.1 ± 3.2	Use of VR in cognitive training at an early phase led to greater improvement in cognitive functioning in stroke patients.	Expensive devices required. Current clinical rehabilitation practices lack a standard training model, and their clinical efficacy has limitations.
Gueye et al., 2021 [22]	Cognitive Function, Functional Abilities in Daily Life, Upper Extremity motor performance, Quality of life, and mood	MoCA	25	25	21.8 ± 4.88	20.3 ± 6.14	25.6 ± 3.54	22.9 ± 5.53	There was no statistically significant difference in cognitive function between intervention and control groups.	Single-center study: Cost-effectiveness of the intervention has not been evaluated
Chatterjee et al., 2022 [23]	Cognitive function, Functional and daily living abilities, Psychological well-being, Quality of Life, Length of Hospital Stay, Treatment acceptability, and safety intervention	MoCA	22	7	MoCA < 15 = 17; MoCA 15-24 = 22	12.5	MoCA < 15 = 17; MoCA 15-24 = 22	10	Significant improvement in overall cognitive function was observed	Assessment was conducted over a short-term period, hence long-term benefits and disadvantages were not properly assessed. No long-term follow-up done. Many patients were lost in the follow-up phase.
Maggio et al., 2025 [24]	Cognitive function, Emotional state, Motivation	MoCA	27	27	25	25	26	25	Significant improvement in cognitive function, as well as motivation and emotional state, in IG as compared to CG	Potential for bias due to self reported measures Neurophysiological measures were not used to directly assess brain plasticity Did not assess effect of level of immersiveness of the VR on patients’ outcomes Did not assess the effect of improved cognitive function on everyday activities Participants with severe cognitive impairment (MoCA ≤ 18) were excluded Doubts about the strength of effect of intervention as only few participants had exceeded the minimum detectable difference threshold (4 points) on MoCA scale.

Table 3 summarizes mental health outcomes that were assessed in five out of the nine RCTs. Various validated tools were used, including: MMSE (n=2), BDI-II (n=1), Nottingham Extended Activities of Daily Living (NEADL), and hospital Anxiety and Depression Scale (HADS) (n=1), as well as HAM-A, HRS-D, and McClelland tests (n=1). Following VR-based intervention, mental health improved over time in both groups in the five studies, with a significant difference observed between the two groups in two of the studies and no statistically significant improvement in the remaining three studies.

**Table 3:** Mental Health Assessment: Tools used and Results

Author, Year	Tools Used to Assess Mental Health of Participants	Results	
			Baltaduonienė et al., 2019 [16]	MMSE	MMSE scores in IG rose from 24.88 ± 2.58 to 26.90 ± 2.56, while CG changed from 22.95 ± 3.52 to 25.13 ± 3.13. Analysis revealed significant differences between the two groups, as demonstrated by a p-value of 0.03.	
Oh et al., 2019 [17]	K-MMSE, mean ± SD	A K-MMSE of 27.3 ± 3.0 was measured in the IG, while that of the CG was 27.9 ± 1.5. Both IG and CG have shown a substantial increase in K-MMSE scores (p=0.038 and p=0.001, respectively) over time.	
Manuli et al., 2020 [18]	Beck Depression Inventory-II (BDI-II) denoted by: pre-treatment score - post-treatment score, mean ± SD	The BDI-II score of IG was 13.0 ± 4.8 - 5.6 ± 3.2, while that of the CG was 10.9 ± 5.2 - 9.8 ± 4.9. There was no significant difference observed between the two groups.	
Chatterjee et al., 2022 [23]	Nottingham Extended Activities of Daily Living (NEADL) and hospital Anxiety and Depression Scale (HADS), median (Interquartile range)	The difference in NEADL score between end of 3 months and baseline in the (VRT (VR training) and usual care in patients with baseline MoCA score < 15) and (VRT and usual care in patients with baseline MoCA score of 15-24) is -6 (-44) and 13 (32), respectively, as compared to 0(22) in the CG. The gap in depression between the end of 3 months and baseline in the (VRT and usual care in patients with baseline MoCA score < 15) and (VRT and usual care in patients with baseline MoCA score of 15-24) is -2.5(7.5) and 0(-4.5), respectively, as compared to 1(3) in the CG. The change in anxiety between end of 3 months and baseline in the (VRT and usual care in patients with baseline MoCA score < 15) and (VRT and usual care in patients with baseline MoCA score of 15-24) is -1(3) and -4(-8.5), respectively, as compared to 1(4.5) in the CG. Anxiety score was decreased significantly in the intervention group with a baseline MoCA score of 15-24.	
			Maggio et al., 2025 [24]	Hamilton Anxiety Rating Scale (HAM-A), Hamilton Depression Rating Scale (HRS-D), and the McClelland test to assess motivation	Significant improvements were observed in HRS-D (p <0.001), McClelland Test-Achievement (p<0.001), McClelland Test-Affiliation (p=0.01) and McClelland Test-Power (0.0002) in the IG. CG did not show significant differences in these measures.	

Table 4 outlines the improvement in patients’ quality of life (QoL), which was assessed using the SF-12 Health Survey (n=1) and EuroQoL (n=1). Significant improvement in QoL was observed in only one of the studies.

**Table 4:** Assessment of Patient’s Quality of Life (QoL): Tools used and Results

Author, Year	Tools Used to Assess Quality of Life (QoL) of the Patients	Results	
			Manuli et al., 2020 [18]	SF-12 (Short Form-12) Health Survey - Score denoted as pre-treatment score - post-treatment score, mean ± SD	The results for IG were 24.7 ± 9.0 - 36.3 ± 10.9, while those of the CG were 28.7 ± 8.0 - 30.1 ± 8.2. Significant improvement in QoL was observed in the intervention group.	
Chatterjee et al., 2022 [23]	Quality of Life (EuroQoL), median (Interquartile range)	The difference in EuroQoL score between the end of 3 months and baseline in the (VRT and usual care in patients with baseline MoCA score < 15) and (VRT and usual care in patients with baseline MoCA score of 15-24) is 0(4) and 0(-7.5), respectively, as compared to CG which was 0 (-2). The authors did not report the presence or absence of any significant difference between the two groups.	

Table 5 presents disability and independence outcomes, assessed in three studies using either the Functional Independence Measure (FIM) (n=2) or the Modified Barthel Index (MBI) (n=1). The three studies showed no statistically significant difference between the intervention and control groups.

**Table 5:** Assessment of Level of Disability: Tools Used and Results

Author, Year	Tools Used to Assess Patients’ Level of Disability and Autonomy in Carrying Out Daily Activities	Results	
			Manuli et al., 2020 [18]	Functional Independence Measure (FIM) - denoted as: pre-treatment score - post-treatment score, mean ± SD	The scores for IG and CG were as follows: 73.1 ± 4.8 - 96.1 ± 7.8 and 47.7 ± 3.6 - 52.9 ± 4.3. Improvement was seen in both, with neither demonstrating a significantly greater benefit over the other group.	
Mao et al., 2020 [19]	Modified Barthel index (MBI), mean ± SD	At baseline, MBI was 33 ± 10 in IG and 34 ± 11 in CG. Following the treatment, these increased to 52 ± 7 and 46 ± 10, respectively. MBI was not statistically different between the two groups.	
Gueye et al., 2021 [22]	FIM, mean ± SD	The IG showed an increase in FIM score from 89.0 ± 14.35 to 110.8 ± 8.17, while the CG changed from 82.8 ± 19.92 to 104.9 ± 15.49. There was no statistically significant difference in disability level between intervention and control groups.	

**Figure 2:**
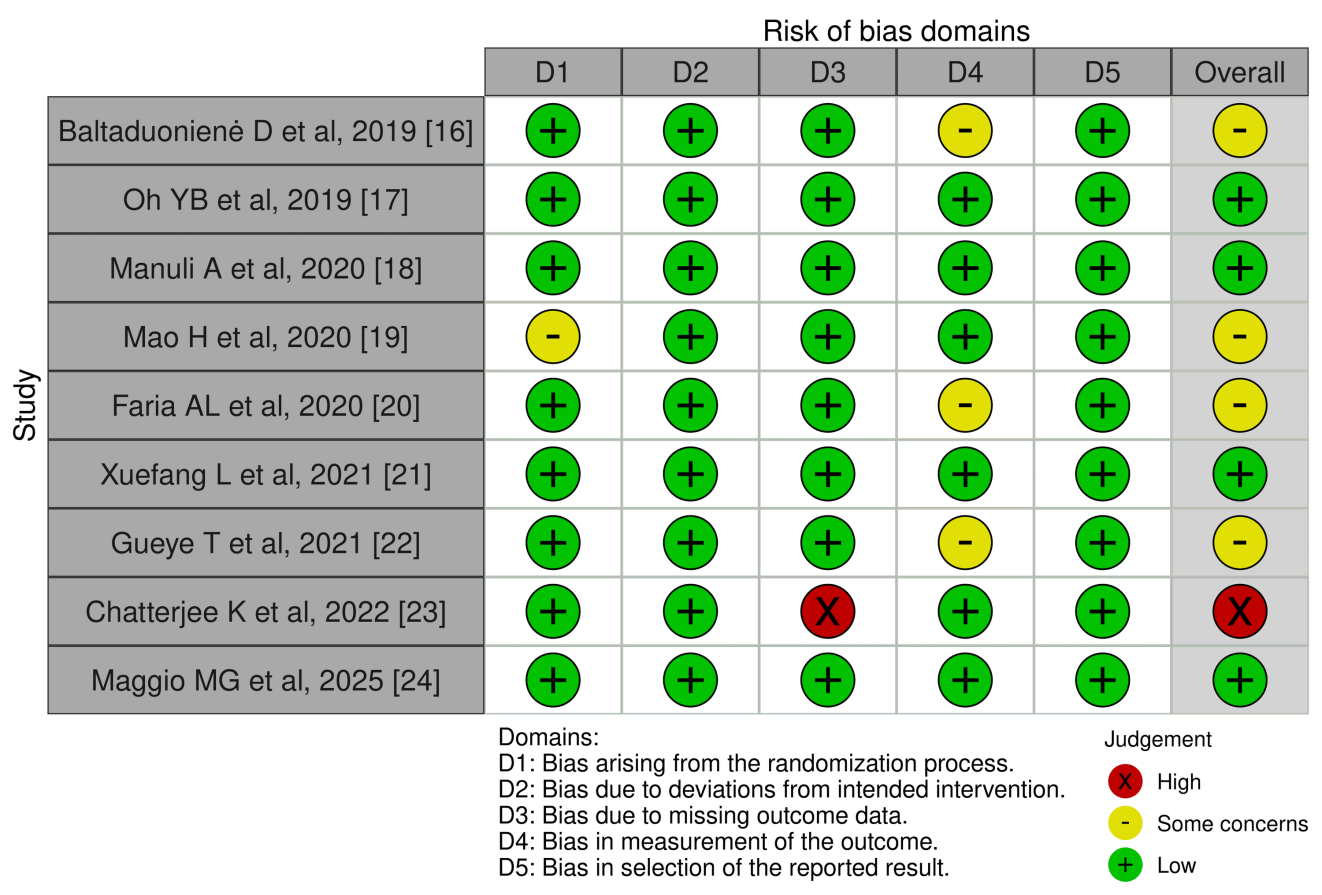
Traffic Light Plot (Risk of Bias Assessment) [16-24]

**Figure 3:**
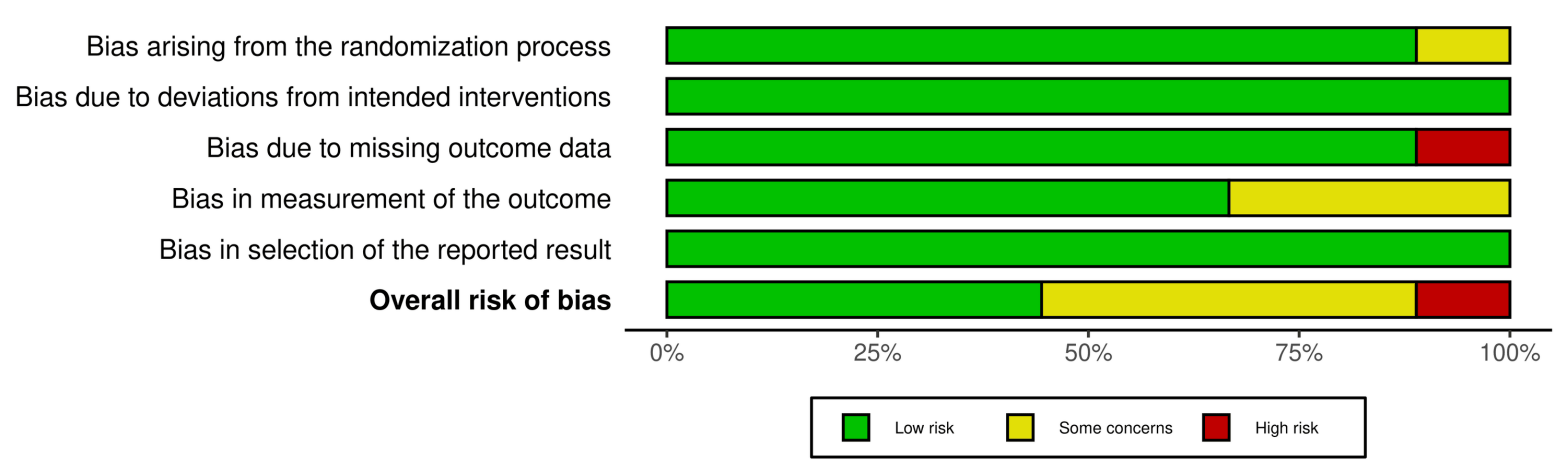
Weighted Bar Plots (Risk of Bias Assessment)

The selected articles were critically appraised, with Figures 2-3 illustrating the results [16-24]. The results of the assessment across the five domains were: bias arising from the randomization process (D1): low risk (88.9%) and some concern (11.1%); bias due to deviations from intended intervention (D2): low risk (100%); bias due to missing outcome data (D3): low risk (88.9%) and high risk (11.1%); bias in measurement of outcome (D4): low risk (66.7%) and some concerns (33.3%); and bias in selection of the reported result (D5): low risk (100%). Accordingly, four of the articles were of some concern, followed by four of low risk and one of high risk.

Discussion

This systematic review evaluated the effectiveness of virtual reality-based cognitive rehabilitation following stroke, based on nine randomized controlled trials [16-24] encompassing 601 patients, and found that seven out of the nine studies reported significant post-intervention gains in MoCA scores in the VR groups compared to the control group, with an average cognitive improvement approximating 12.8%. It was also seen that immersive or combined VR-based interventions were associated with greater improvements in cognitive function, with the most pronounced positive effects observed in interventions that combined VR with robotics and personalized feedback. Significant secondary outcomes such as mental health, QoL, and functional independence were also assessed in some of the studies. Mental health displayed significant improvement in the five studies that evaluated them, while QoL was assessed in two studies, with one showing positive trends following VR-based therapy, and three studies reported improved scores on measures of functional independence and disability. The above findings evidenced the multidimensional potential of VR-based therapy.

Comparative analysis of the nine included RCTs revealed that despite the common goal of improvement of post-stroke cognitive impairment via use of VR-based therapy, each trial employed interventions of varying characteristics that ultimately affected the end result. For example, Manuli et al. (2020) uniquely combined robotic-assisted gait training with VR and were able to demonstrate significant cognitive and functional improvement that resulted in a shorter recovery period [18]. Synergism of motor and cognitive rehabilitation therapy was also explored by Mao et al., where integrated mirror neuron-based training with conventional upper limb therapy was used and yielded positive results [19]. Another distinct feature of some interventions was the immersive VR therapy explored by Faria et al. with Reh@City v2.0, a VR platform designed to mimic real-life tasks, and Chatterjee et al (2022, with VIRTUE, where significantly positive outcomes were observed [20, 23]. VIRTUE, in contrast to Reh@city, also proved beneficial to patients with severe cognitive deficits [20, 23]. Non-immersive VR therapy tested by Oh et al. and Baltaduonienė et al. resulted in modest gains in cognitive function that may be due to limited immersion or shorter intervention [16, 17]. Semi-immersive VR therapy, explored by Maggio et al., with the VRSS that also combined emotional and motivation assessments, allowing for broader assessment of the rehabilitation process and overwhelmingly positive outcomes in terms of cognition, as assessed by the MoCA, once again displayed the superiority of combined therapy in the case of rehabilitation [24]. The remaining study yielding a positive outcome was Xuefang et al., which combined early-phase VR therapy with acupuncture, an approach that led to significant MoCA gains and suggested potential neurophysiological augmentation [21]. Finally, Gueye et al. were among the few to examine acute stroke populations but showed no significant difference, hence highlighting that timing alone may not suffice in adequately addressing deficits [22].

Although the above analysis allows emphasis on common trends among studies, a formal meta-analysis was not possible due to large variation in intervention design, outcome reporting methods, and failure to report consistent statistical parameters like effect sizes, 95 % confidence intervals, and p-values in many of the original articles. There were also many methodological limitations, such as the overall medium quality of evidence, the majority of studies being single-center trials with small sample sizes, short follow-up durations, and the exclusion of patients with severe cognitive impairment. These limitations reduce the statistical power and do not represent the total stroke population having cognitive impairment. Furthermore, it is to be noted that there is a lack of stratified analysis by demographic variables such as age, sex, or socioeconomic status. Another significant oversight was that none of the studies reported the influence of health insurance status or cost burden, which are important considerations given the high cost and limited accessibility of VR technologies, especially in low and middle-income countries. As suggested by the ROB 2 analysis, the trial conducted by Chatterjee K et al, 2022 [23], showed high concern due to missing outcome data. Studies showing some concern were mainly due to faulty measurement of the outcome or bias arising from the randomization process. Additionally, low-risk studies, that is, 2020, Oh et al., Manuli et al., Xuefang et al., and Maggio et al. 2025 have high reliability and strong internal reliability [17,18,21,24].

Clinical implications from this review suggest that VR would prove a valuable addition to standard post-stroke rehabilitation as it offers both cognitive and functional benefits. However, wider adoption of therapy requires tackling issues pertaining to cost and standardization of treatment protocols. Future research on the topic should hence prioritize larger, multi-center trials with longer follow-up periods, comprehensive subgroup analyses, and economic evaluations to determine the cost-effectiveness and true capacity of VR-based rehabilitation, especially in diverse healthcare settings.

## Conclusions

In this systematic review, virtual reality-based cognitive rehabilitation therapy consistently yielded superior neurocognitive gains compared with standard therapy. Additional benefits were observed in mental health, QoL, and functional independence, suggestive of VR’s broader therapeutic potential. While conventional therapy remains the mainstay for post-stroke neurocognitive recovery, integration of VR exercises could accelerate patient progress owing to their interactive nature as well as good patient acceptability. But it is to be noted that future trials focusing on cost-effective, accessible, and patient-tailored VR interventions are required to establish the long-term efficacy of these tools before their application in the mainstream.

## References

[1] (2025). Institute for Health Metrics and Evaluation (IHME): Global Burden of Disease 2021: Findings from the GBD 2021 Study. Global Burden of Disease.

[2] Feigin VL, Brainin M, Norrving B (2025). World Stroke Organization: Global Stroke Fact Sheet 2025. Int J Stroke.

[3] Ozkan H, Ambler G, Esmail T, Banerjee G, Simister RJ, Werring DJ (2025). Prevalence, trajectory, and factors associated with patient-reported nonmotor outcomes after stroke: A systematic review and meta-analysis. JAMA Netw Open.

[4] Melkas S, Jokinen H, Hietanen M, Erkinjuntti T (2014). Poststroke cognitive impairment and dementia: Prevalence, diagnosis, and treatment. Degener Neurol Neuromuscul Dis.

[5] Jeffares I, Rohde D, Doyle F, Horgan F, Hickey A (2022). The impact of stroke, cognitive function and post-stroke cognitive impairment (PSCI) on healthcare utilisation in Ireland: A cross-sectional nationally representative study. BMC Health Serv Res.

[6] Khan A, Imam YZ, Muneer M, Al Jerdi S, Gill SK (2024). Virtual reality in stroke recovery: A meta-review of systematic reviews. Bioelectron Med.

[7] Gangemi A, De Luca R, Fabio RA (2023). Effects of virtual reality cognitive training on neuroplasticity: A quasi-randomized clinical trial in patients with stroke. Biomedicines.

[8] Laver KE, Lange B, George S, Deutsch JE, Saposnik G, Crotty M (2017). Virtual reality for stroke rehabilitation. Cochrane Database Syst Rev.

[9] Gao Y, Ma L, Lin C (2021). Effects of virtual reality-based intervention on cognition, motor function, mood, and activities of daily living in patients with chronic stroke: A systematic review and meta-analysis of randomized controlled trials. Front Aging Neurosci.

[10] Rose Sin Yi L, Jing Jing S, Hammoda AO, Jonathan B, Ladislav B, Jing Q (2024). Effects of virtual reality-based cognitive interventions on cognitive function and activity of daily living among stroke patients: Systematic review and meta-analysis. J Clin Nurs.

[11] Chiti G, Pantoni L (2014). Use of Montreal Cognitive Assessment in patients with stroke. Stroke.

[12] Moher D, Liberati A, Tetzlaff J, Altman DG (2009). Preferred reporting items for systematic reviews and meta-analyses: The PRISMA statement. PLoS Med.

[13] Ouzzani M, Hammady H, Fedorowicz Z, Elmagarmid A (2016). Rayyan-a web and mobile app for systematic reviews. Syst Rev.

[14] Sterne JA, Savović J, Page MJ (2019). RoB 2: A revised tool for assessing risk of bias in randomised trials. BMJ.

[15] McGuinness LA, Higgins JP (2021). Risk-of-bias VISualization (robvis): An R package and Shiny web app for visualizing risk-of-bias assessments. Res Synth Methods.

[16] Baltaduonienė D, Kubilius R, Berškienė K, Vitkus L, Petruševičienė D (2019). Change of cognitive functions after stroke with rehabilitation systems. Transl Neurosci.

[17] Oh YB, Kim GW, Han KS, Won YH, Park SH, Seo JH, Ko MH (2019). Efficacy of virtual reality combined with real instrument training for patients with stroke: A randomized controlled trial. Arch Phys Med Rehabil.

[18] Manuli A, Maggio MG, Latella D (2020). Can robotic gait rehabilitation plus Virtual Reality affect cognitive and behavioural outcomes in patients with chronic stroke? A randomized controlled trial involving three different protocols. J Stroke Cerebrovasc Dis.

[19] Mao H, Li Y, Tang L, Chen Y, Ni J, Liu L, Shan C (2020). Effects of mirror neuron system-based training on rehabilitation of stroke patients. Brain Behav.

[20] Faria AL, Pinho MS, Bermúdez I Badia S (2020). A comparison of two personalization and adaptive cognitive rehabilitation approaches: a randomized controlled trial with chronic stroke patients. J Neuroeng Rehabil.

[21] Xuefang L, Guihua W, Fengru M (2021). The effect of early cognitive training and rehabilitation for patients with cognitive dysfunction in stroke. Int J Methods Psychiatr Res.

[22] Gueye T, Dedkova M, Rogalewicz V, Grunerova-Lippertova M, Angerova Y (2021). Early post-stroke rehabilitation for upper limb motor function using virtual reality and exoskeleton: equally efficient in older patients. Neurol Neurochir Pol.

[23] Chatterjee K, Buchanan A, Cottrell K, Hughes S, Day TW, John NW (2022). Immersive virtual reality for the cognitive rehabilitation of stroke survivors. IEEE Trans Neural Syst Rehabil Eng.

[24] Maggio MG, Bonanno L, Rizzo A (2025). The role of virtual reality-based cognitive training in enhancing motivation and cognitive functions in individuals with chronic stroke. Sci Rep.

